# Overcoming the Difficulties of Cohort Studies

**DOI:** 10.2188/jea.JE20120225

**Published:** 2013-05-05

**Authors:** Kazunori Kayaba

**Affiliations:** Saitama Prefectural University, Koshigaya, Saitama, Japan

A cohort study is a longitudinal observational study of exposure and outcome status. Cohort studies are associated with well-known disadvantages.

Some studies are able to easily recruit subjects and can be finished within 2 hours, eg, asking whether students who skipped breakfast are more likely than those who ate breakfast to fall asleep during a lecture. However, many cohort studies require a large sample size and long follow-up period because of the low incidence and long latency periods for outcomes, as is the case with malignant neoplasms, atherosclerotic cardiovascular disease, and dementia. Consequently, the study becomes complex and costly. Researchers must also contend with the harsh realities of cohort study management. Although many articles have described cohort study designs, few have explained how to manage this type of investigation.

In an article on the Japan Collaborative Cohort Study published in this issue, Tamakoshi and colleagues touch on some of the serious obstacles they encountered in conducting that study: retirement of major researchers, city mergers in the year 2000, and reduced funding.^1^ In a community-based, large-scale cohort study, a significant resource investment is required for the baseline examination and consequent follow-up surveys, which take more than 10 years in many studies.

The cohort study design requires a multidisciplinary team, including physicians, public health nurses, public officials, and representatives of community residents. The many stakeholders belong to different organizations and have diverse areas of responsibility. Therefore, a well-organized network is necessary to ensure effective collaboration among team members. These requirements demand research leaders who are knowledgeable about the administration of complex systems. One scheme for facilitating collaboration between disciplines is interprofessional work,^2^ and the importance of this type of education in undergraduate and continuing medical education has been widely recognized.^3^

In Japan, payment for full-time workers is not usually covered by government research grants. Resources and aid for a study largely depend on support from the various organizations involved in the study, such as local governments, private companies, and nonprofit organizations. Researchers must produce study results each year, although the final outcome of a cohort study will not be available for many years. This can result in conflicting expectations between the researchers and executive officers of supporting foundations or organizations. Uncertainty regarding financing creates doubt among researchers as to whether they will be able to finish a study project. Epidemiology associations should continue to appeal to governmental and private organizations for understanding of the characteristics of a cohort study and for long-term support.

The number of municipalities in Japan has decreased by 47%, from 3229 in 1999 to 1719 in 2012, according to a report by the Ministry of Internal Affairs and Communications. Many community-based cohort studies are based on health-promotion activities sponsored by Japanese local governments. Thus, these mergers could dramatically change study communities, and major restructuring of local government offices could interfere with the conduct of studies. Not all merging municipalities share an enthusiasm for cohort studies. In addition, mergers force researchers to reconstruct research teams, and may result in duplication of effort.

Because long-term cohort studies need to continue for more than a decade, retirement of senior researchers is an unavoidable issue. Even though new researchers may have confidence in the scientific validity of the study hypotheses, they might feel that participation in a cohort study is likely to cause an academic career crisis because it is uncertain if the final outcome will be important.

Tamakoshi and colleagues raise awareness of the difficult issues involved in the conduct of long-term, large-scale, community-based, multi-site cohort studies. Unfortunately, these issues appear to be far from resolved. However, a large community-based cohort study can yield strong evidence for a causal relationship between an exposure and an outcome, and may even allow for estimation of effect size.^4^ One well-known cohort study succeeded in establishing excellent relationships with a targeted community and recruited young investigators to become involved in long-term projects.^5^

To overcome the difficulties of conducting cohort studies, epidemiologists must continue to speak and write about the scientific importance of cohort studies and the public health significance of the outcomes revealed by such investigations.
